# Construction and Modelling of an Inducible Positive Feedback Loop Stably Integrated in a Mammalian Cell-Line

**DOI:** 10.1371/journal.pcbi.1002074

**Published:** 2011-06-30

**Authors:** Velia Siciliano, Filippo Menolascina, Lucia Marucci, Chiara Fracassi, Immacolata Garzilli, Maria Nicoletta Moretti, Diego di Bernardo

**Affiliations:** 1 Telethon Institute of Genetics and Medicine (TIGEM), Naples, Italy; 2 Department of Computer and Systems Engineering, Federico II University, Naples, Italy; Duke University, United States of America

## Abstract

Understanding the relationship between topology and dynamics of transcriptional regulatory networks in mammalian cells is essential to elucidate the biology of complex regulatory and signaling pathways. Here, we characterised, via a synthetic biology approach, a transcriptional positive feedback loop (PFL) by generating a clonal population of mammalian cells (CHO) carrying a stable integration of the construct. The PFL network consists of the Tetracycline-controlled transactivator (tTA), whose expression is regulated by a tTA responsive promoter (*CMV-TET*), thus giving rise to a positive feedback. The same *CMV-TET* promoter drives also the expression of a destabilised yellow fluorescent protein (d2EYFP), thus the dynamic behaviour can be followed by time-lapse microscopy. The PFL network was compared to an engineered version of the network lacking the positive feedback loop (NOPFL), by expressing the *tTA* mRNA from a constitutive promoter. Doxycycline was used to repress tTA activation (switch off), and the resulting changes in fluorescence intensity for both the PFL and NOPFL networks were followed for up to 43 h. We observed a striking difference in the dynamics of the PFL and NOPFL networks. Using non-linear dynamical models, able to recapitulate experimental observations, we demonstrated a link between network topology and network dynamics. Namely, transcriptional positive autoregulation can significantly slow down the “switch off” times, as comparared to the non
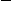
autoregulatated system. Doxycycline concentration can modulate the response times of the PFL, whereas the NOPFL always switches off with the same dynamics. Moreover, the PFL can exhibit bistability for a range of Doxycycline concentrations. Since the PFL motif is often found in naturally occurring transcriptional and signaling pathways, we believe our work can be instrumental to characterise their behaviour.

## Author Summary

Synthetic Biology aims at designing and building new biological functions in living organisms. At the same time, Synthetic Biology approaches can be used to uncover the design principles of natural biological systems through the rational construction of simplified regulatory networks. Mathematical models of the networks are then derived from physical considerations and can be used to explain the observed dynamical behaviours. We have characterised a regulatory motif often found in transcriptional and signalling pathways. We constructed a positive feedback loop motif in mammalian cells, consisting of a protein controlling its own expression. We have shown that this motif exhibits a dynamic behaviour which is very different from that obtained when the autoregulation is removed. This difference is intrinsic to the specific wiring diagram chosen by the cell to control its behaviour (feedback versus non-feedback configurations), and can be instrumental in understanding the complex network of regulation occurring in a cell.

## Introduction

Synthetic biology can be used to uncover the design principles of natural biological systems through the rational construction of simplified regulatory networks [1]. So far, it has been shown that feedback and feed-forward loops are essential regulatory motifs involved in transcriptional regulation [2]. For example, natural circadian clocks consist of feedback loops in which a set of transcriptional activators and repressors are connected by mutual feedback; these clocks are able to maintain a 24-h periodicity in protein expression [3]. How exactly this is achieved is still under debate, but it is an intrinsic property of the network topology [4]. Therefore, understanding the relationship between topology and dynamics of transcriptional regulatory networks in mammalian cells is essential to elucidate the biology of complex regulatory and signaling pathways.

**Figure 1:**
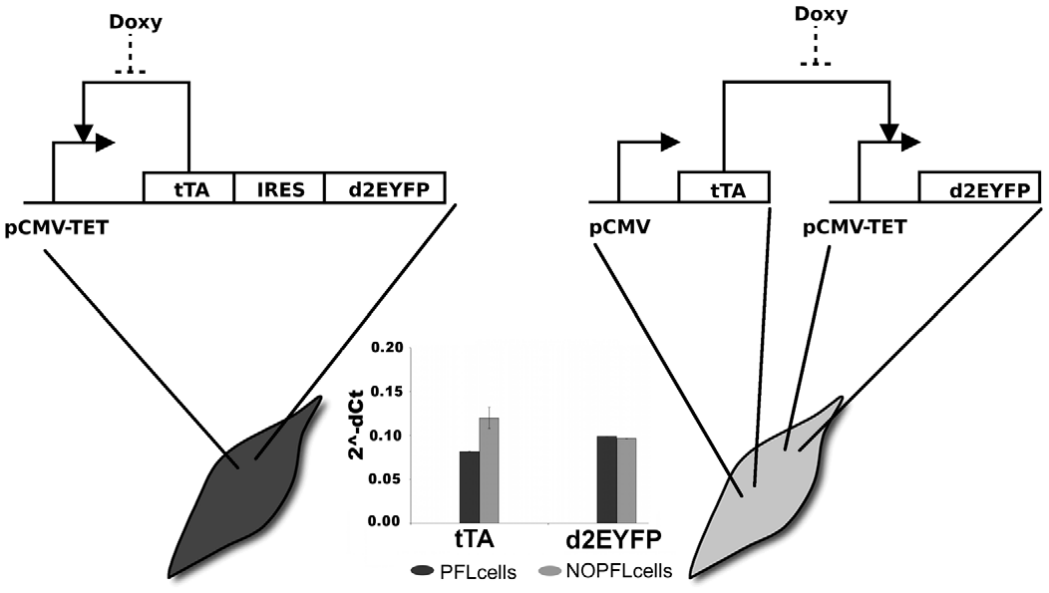
Design of the expression system. (A) PFL: the promoter *CMV-TET* consists of seven direct repeats of a 42-bp sequence containing the tet operator sequences (tetO), located just upstream of the minimal *CMV* promoter (PminCMV). The Tetracycline-controlled transactivator tTA derives from the addition of the VP16 activation domain to the transcriptional repressor TetR. The d2EYFP is the destabilised yellow-green variant of enhanced green fluorescent protein. (B) NOPFL: the *CMV* promoter drives the expression of the tTA, which in turns drives the transcription of the d2EYFP from the *CMV-TET* promoter. (Inset) RealTime PCR performed on DNA extracted from PFL and NOPLF cells shows that the DNA levels of tTA and d2EYFP are comparable among the two clonal cell populations.

In this work, we aimed at characterising an inducible transcriptional positive feedback loop (PFL) in mammalian cells consisting of the *CMV-TET* promoter, responsive to the Tetracycline-controlled transactivator tTA, driving expression of the tTA protein itself and of a fluorescent reporter protein (Fig. 1A). Most of the studies carried out so far in mammalian cells are based on plasmid transfection, which prevents precise quantitative measurements due to the unpredictable amount of plasmids that enters each cell, and to the transient nature of transfection. To overcome these limitations, we generated a clonal population of Chinese Hamster Ovary cells (CHO) carrying a stably integrated version of the PFL, which were then used to evaluate the dynamical properties of this transcriptional motif.

We also performed control experiments by generating a clonal population of cells lacking the positive feedback loop (NOPFL), as shown in Fig. 1B.

In vivo quantitative measurements of fluorescence intensity in time, following addition of the inducer molecule (Doxycyline) able to”switch off” the network, were used to fit an Ordinary Differential Equations (ODE) model of the PFL and NOPFL networks.

The models were able to reproduce the experimental data, and, interestingly, highlighted differences in the dynamic properties of the PFL versus the NOPFL networks, which are due to the intrinsic differences in the two network topologies.

It has been suggested theoretically that the PFL motif can slow down response times of transcriptional regulatory networks [5]. Here, we experimentally demonstrated that this is the case, and we reported for the first time a quantitative characterisation of a transcriptional positive feedback loop in mammalian cells, which can be instrumental in better understanding the properties of natural occurring transcriptional and signaling networks.

## Results

### Construction of the inducible positive feedback loop (PFL) and of the corresponding control network (NOPFL)

Our approach is based on the use of well known and characterised regulators of gene expression, in order to achieve a complete control of the network behaviour. The positive feedback loop (PFL) is shown in Fig. 1A. In particular, we took advantage of the inducible Tet regulatory system; the expression of Tetracycline-controlled transactivator tTA is self-controlled by a *CMV-TET* promoter, responsive to the tTA itself unless the Tetracycline, or its analogous Doxycycline, is added to the medium in which cells are grown [6]. To follow the dynamics of the PFL, a destabilised yellow-green variant of the enhanced green fluorescent protein (d2EYFP) (Clontech), with a reported half-life of approximately two hours, had to be placed under the control of the same promoter. To this end, we constructed a unique cassette with an Intra Ribosomal Entry Sequence (IRES) in between of the transactivator tTA and the d2EYFP, which enables a single mRNA to encode for two different proteins (Fig. 1A).

In order to stably express in CHO cells the PFL network, and to generate a clonal population, we inserted the cassette in Fig. 1A in a lentiviral vector [7] [8]. Infected cells were first sorted by Fluorescence Activated Cell Sorter (FACS) and then a clonal population of CHO cells carrying the PFL was generated by single cell expansion (PFL cells) (Experimental procedure: cell culture, lentiviral transduction, switch-off experiment).

To capture the dynamic properties intrinsic to the PFL, we needed to generate a control network lacking the positive feedback loop (NOPFL), but using the same biological “parts” as in the PFL network. As shown in Fig. 1B, we constructed a cassette containing the same *CMV-TET* promoter upstream of the d2EYFP. The tTA protein, this time, was placed under the control of a constitutive promoter, thus breaking the feedback loop. Using the same strategy described above, we generated a clonal population of CHO cells carrying the NOPFL network (NOPFL cells). We experimentally verified that both PFL and NOPFL cells have the same number of tTA/d2EYFP DNA integrations (Fig. 1 inset). (Experimental procedure: DNA extraction, RealTime PCR).

### Experimental determination of the reporter protein degradation

**Figure 2:**
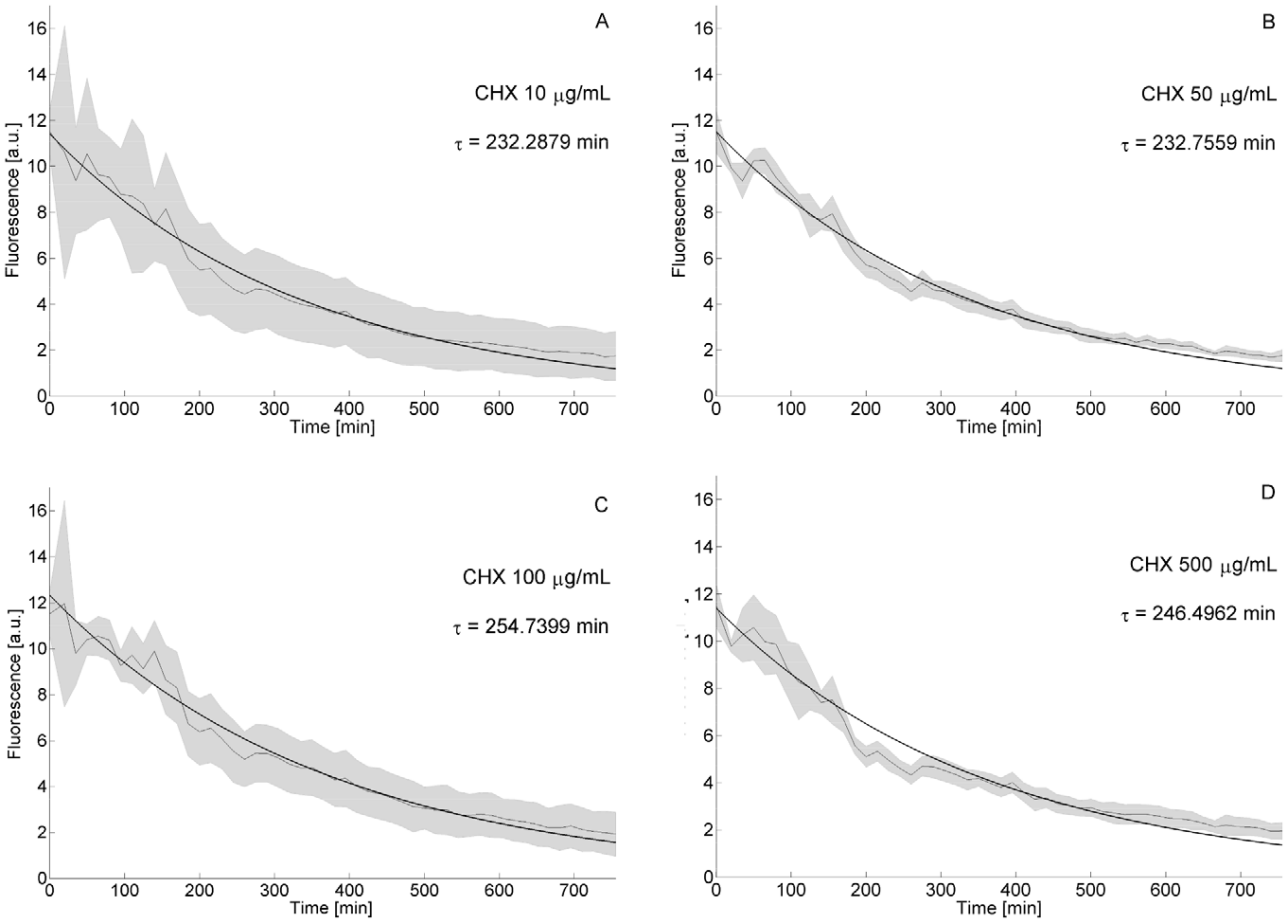
Degradation kinetics of d2EYFP. *CMV-TET*-d2EYFP stably integrated CHO AA8 TET-OFF cells were treated at t = 0 with different concentrations of Cyclohexamide (CHX): panel A, 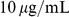; panel B, 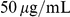; panel C, 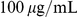; panel D, 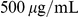. Fluorescence intensity was followed up to 750 minutes. Sampling time is equal to 15 min. The thin line represents the mean over biological triplicates; the shaded area represents the standard error. Experimental data were used to fit the exponential decay of d2EYFP protein levels, and thus to derive its half-life (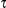). Fluorescence intensity in untreated cells was not subjected to any significant decay (data not shown).

We experimentally evaluated the degradation rate of the reporter protein (d2EYFP) since this is a key parameter which affects the observed fluorescence dynamics. To evaluate d2EYFP degradation rate, stably integrated NOPFL cells were treated with Cyclohexamide to a final concentration of 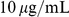, 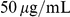, 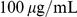 or 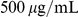, to inhibit protein synthesis [9]. The fluorescence intensity of NOFPL cells was followed for 12 hrs and images were acquired at 15 min intervals. The resulting d2EYFP dynamics are shown on Fig. 2 and appear very similar, independently of the Cyclohexamide concentrations. The experimental data were fitted to an exponential curve 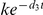, and the degradation coefficient 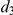 was used to obtain the half-life (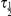) of the d2EYFP protein: 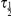 = log(2)/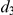 (Fig. 2 and Table 1). We estimated 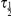 to be in the range 3.6 h–4.4 h. (Experimental procedure: determination of d2EYFP half-life). The estimated value is about two-fold the reported d2EYFP half-life of 2 h [10]; we believe that this discrepancy is likely due to the fact that cells were grown at a temperature 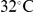, rather than the usual 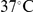.

**Table 1:** Parameters identified after the fitting procedures: parameters values as well as standard deviation are reported for each parameter.

Parameter	Description	Fitted value	STD
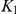 [nM]	Activation coefficient	4.81	1.06
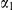	Basal activity CMV-TET promoter	1.13E-05	3.62E-05
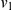 [nM min 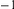 ]	Maximal transcription rate CMV-TET promoter	7.54E-02	1.97E-02
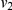 [min 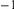 ]	General translation rate	2.71E-02	1.22E-02
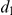 [min 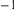 ]	Degradation rate tTA mRNA	1.01E-02	1.22E-03
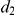 [min 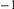 ]	Degradation rate tTA protein	1.00E-02	3.42E-03
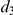 [min 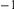 ]	Degradation rate d2EYFP protein	3.24E-03	2.66E-04
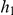	Hill coefficient of the CMV-TET promoter	3.16	1.40E-01
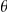 [nM]	Affinity Doxycycline - CMV-TET promoter interaction	1.00	8.85E-03
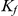 [min 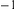 ]	Folding rate d2EYFP	1.24E-03	1.41E-02
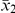 [nM]	Steady state tTA in NOPFL	13.69	7.63E-01
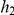	Hill coefficient for Doxycyline	6.03E-02	7.19E-03

### Experimental investigation of the dynamic behaviour of the PFL and NOPFL networks

To observe the dynamics of the PFL and NOPFL networks, we performed time-series experiments in which stably-integrated CHO-PFL cells and CHO-NOPFL cells were imaged using time-lapse fluorescence microscopy (Experimental procedure: cell culture, lentiviral transduction, switch-off experiment). The experimental design consisted in treating both PFL and NOPFL cells with different amounts of Doxycycline in order to “switch off” the circuit, by preventing the tTA protein from binding the *CMV-TET* promoter. We tested the following Doxycyline concentrations: 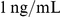, 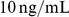, 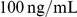 and 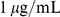 and followed the dynamic behaviour of both the PFL and NOPFL cells for 43 h, collecting images every 15 min, and quantifying the average fluorescence intensity of the cell population (Image acquisition and processing). In this way, we averaged out cell-to-cell variability in the response, since at the beginning of each experiment the tracked microscopy field contained at least 15 cells.

**Figure 3:**
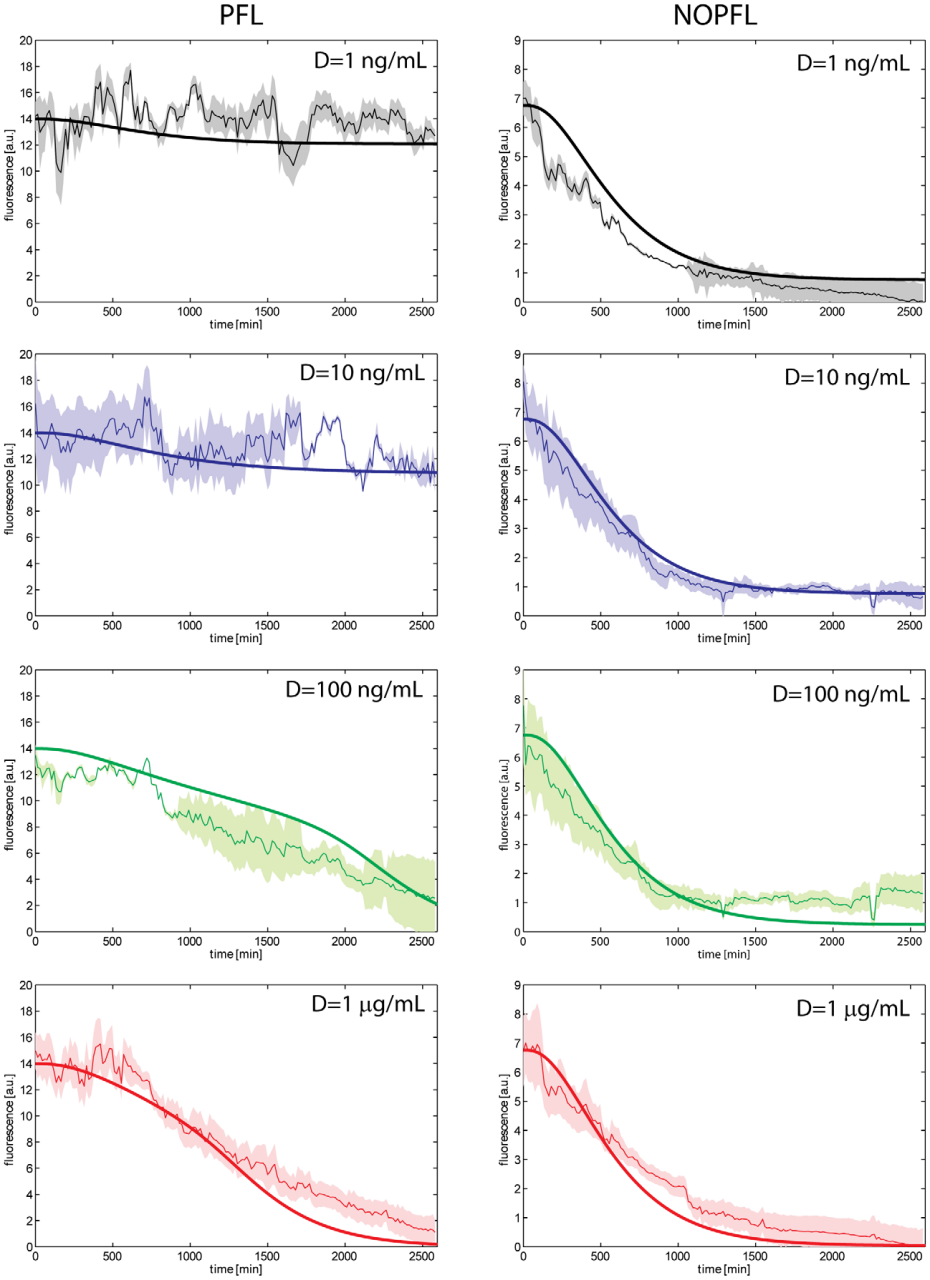
Experimental and simulated switch off time-course across the PFL and NPFL cell population. Experimental data (thin lines) and model simulations (thick lines) were reported for the PFL (left) and NOPFL (right) cells. Shaded areas represent standard deviations from replicate experiments.

**Figure 4:**
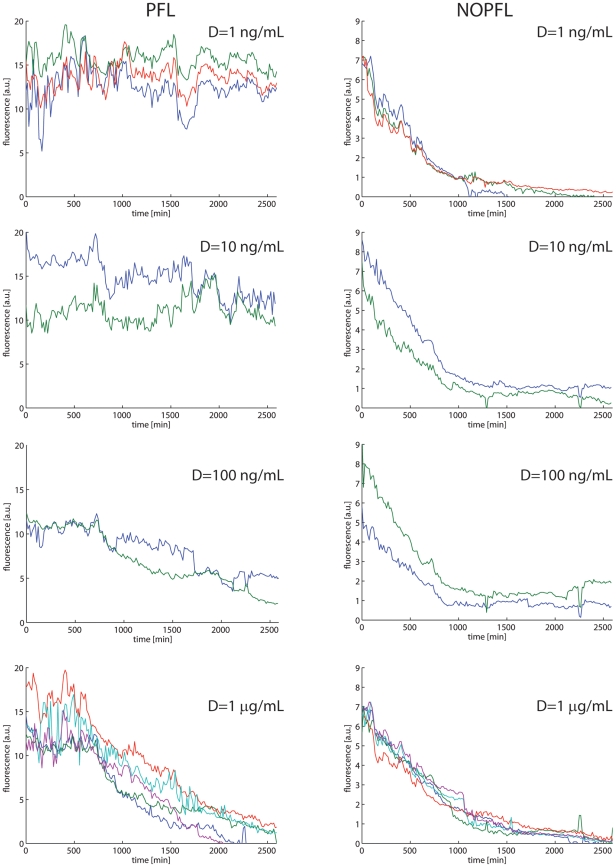
Replicates of the experimental time-courses across the PFL and NPFL cell population. Replicates of the experimental time-courses for the PFL (left) and NOPFL (right) cells. Each line in each panel represent the average fluorescence intensity across the cell population in one switch-off experiment.

Experiments were carried out at a temperature of 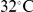 in order to limit cell motility and reduce the risk associated to data loss occurring when cells exit the tracked field [11]. The average fluorescence intensity of the reporter gene across the cell population for both the PFL and NOPFL networks is shown in Fig. 3 for the different concentrations of Doxycycline indicated. In Fig. 4 replicate time-course experiments are shown for each of the Doxycycline concetrations used.

The most striking feature is the slow down in the switch off time of the PFL cells as compared to the NOPFL cells; moreover the switch off time of the PFL is affected by Doxycycline concentrations, whereas NOPFL cells always switch off with approximately the same dynamics. We derived a model of the PFL and NOPFL networks using Ordinary Differential Equations (ODEs). ODEs are commonly used to describe the average behaviour of a population of cells [12] and have been shown to be appropriate for the analysis of synthetic networks in a number of cases ([13], [14], [15], [16], [17], [18]). In our settings, the use of such a modelling approach is valid, since we are measuring the average behaviour of a clonal population of cells.

For each species, i.e. each mRNA and correspondent protein concentration, we wrote one equation, which expresses the change in concentration of the species in a given time interval, as the result of a production term and a degradation term. We assumed:

- Hill functions to model the rate of gene transcription, including basal activity to describe the leakiness of the *CMV-TET* promoter;
- linear degradation for all genes and proteins;
- linear dynamics for the translation;
- Hill functions to model the effect of the inducer (Doxycycline);
- distinct dynamics for the unfolded (inactive) and folded (active) forms of the reporter protein (d2EYFP).

The last assumption was introduced in order to take into account d2EYFP maturation time needed for correct protein folding [16]. Thus, we introduced two differential equations as in [16]: one for the translation of mRNA to the unfolded d2EYFP protein, and one for the folded protein d2EYFP.

Letting 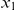 be the *tTA* IRES *d2EYFP* mRNA concentration, 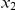 the tTA protein concentration, 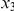 the unfolded d2EYFP protein concentration and 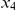 the folded d2EYFP protein concentration, the PFL network can be described as follows:
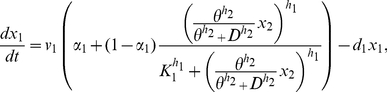
(1)

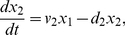
(2)

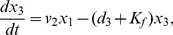
(3)

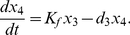
(4)Note that, due to the presence of the IRES sequence, the concentrations of tTA protein and d2EYPF protein depend on the same variable (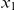), that is the concentration of the single mRNA transcript encoding for both proteins.

For the NOPFL network, we let 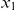 represent only the *d2EYFP* mRNA concentration, and we assumed a constant level (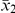) of the tTA protein, due to the constitutive promoter driving 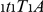 expression in the NOPFL cells. The equations thus become:
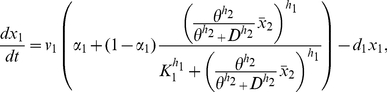
(5)

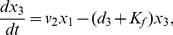
(6)

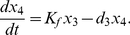
(7)


### Derivation of the model parameters

The parameter estimation problem can be defined as an optimisation problem, where the goal is to minimise a performance measure defined as the error between model predictions and observations, which in our case are the time-series during the “switch off” experiments in Fig. 3 and 4 (Model simulations and parameter identification). In our setting, there are 12 parameters to be fitted, 11 of which are common to both the PFL and NOPLF models (Table 1).

Several alternative strategies can be pursued in order to obtain best estimates of the model parameters, ranging from Newton's method to Genetic Algorithms (GAs). In this work we employed the Trust Region method (TRM) implemented in PottersWheel [19]; thanks to the multi
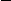
model and multi
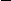
experimental capabilities of this tool, we were able to identify the 12 parameters by simultaneously fitting Eqs. (1) to (7) to all of the experimental time-courses at once. These time-courses include all of the different Doxycycline concentration for both the PFL and NOPFL cells for a total of 24 time-courses, when taking experimental replicates into account.

In order to estimate confidence intervals for each parameter, we run multiple times the TRM identification procedures on the same experimental time-series data, using parameter perturbation routines to allow extensive sampling in the parameters' space.

In order not to introduce any specific bias in our search, we only set admissible ranges for the parameter values to be identified, which reflected physical and biological feasibility, either obtained from literature [15] or directly estimated (degradation rate of 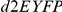 = 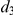). The result of the parameter fitting procedure are reported in Table 1 along with the estimated standard deviation, which are in most of the cases one order of magnitude less than the corresponding parameter value, or at most of the same order of magnitude.

We observed that the paramter 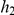 in Table 1, which affects the strength of Doxycycline repression on the tTA protein activity, is much smaller than 1. Usually Hill coefficients are greater than 1, therefore we wondered what could be a possible biological explanation for this behaviour. We observed that for the range of Doxycycline concetration used in the experiment (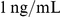 to 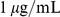), using the parameters' values in Table 1, the function: 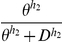 can be approximated by the function: 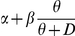 (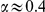 and 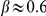). This means that a Michealis-Menten function can also describe the effect of Doxycyline on tTA acitivity, but a certain level of leakiness (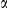) must be taken into account; that is even for large concentrations of Doxycycline, the activity of the tTA protein cannot be completely shut down.

The “switch off” time-series experiments were simulated with both the PFL and NOPFL models using the fitted parameters as shown in Fig. 3. The inferred models are able to recapitulate the observed dynamics in response to different inducer concentrations and experimental settings. Observe that the parameters for both the PFL and NOPFL models are identical, except for 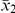 in the NOPFL equations, which is not present in the PFL model. Hence, the observed differences in the dynamical behaviour of the PFL and NOPFL networks are due to the intrinsic differences in their topology, and are robust to changes in parameters values, as demonstrated in the next section.

### Dynamic properties of the PFL and NOPFL networks

In order to further investigate the relationship between topology and dynamical properties, we first observed that the NOPFL model described by Eq.5–7 is a system of linear time-invariant ODEs, for which the theory of liner dynamical systems applies [20]. From the theory, we know that changes in Doxycycline concentration in Eq.5 will not affect the dynamic behaviour of the model, which is governed by the smallest among three degradation terms 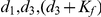 (Model simulations and parameter identification). The concentration of Doxycycline affects only the steady-state values, i.e. how much the network will switch off, but not its dynamics, i.e. how fast it will switch off. Therefore, independently of the values of the parameters, the model of the NOPFL network predicts that for any concentration of Doxycycline, the network will switch off with the same dynamics, albeit possibly reaching different steady-state levels.

**Figure 5:**
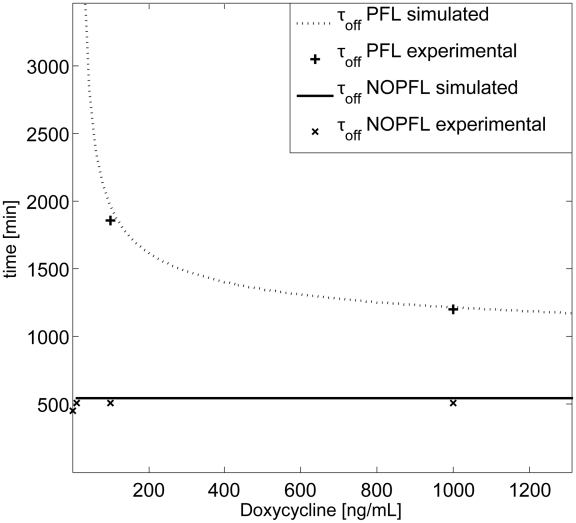
Switch off time 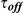 for varying Doxycycline concentrations from experimental data and model predictions. The model predictions for the switch off times 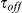 are shown for PFL (dashed thick line) and NOPFL (solid line). Experimental quantification of the 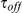 for PFL and NOPFL models have been reported for comparison with + and 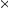 respectively. Observe that the experimental 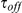 for the PFL at 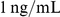 and 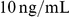 could not be estimated since the PFL is not switching off in the experimental observation time (43 h).

Fig. 5 reports the “switch off” time, 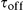, for both the PFL (dashed) and the NOPFL (solid) networks as a function of Doxycycline concentration, computed via numerical simulations of the two models with the parameters estimated in Table 1. 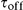 is defined as the time taken by the fluorescence intensity to reach 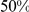 of its final steady-state value (OFF), following treatment with Doxycycline at a given concentration (Material and Methods). As expected, the 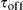 for the NOPFL network is constant and does not change with Doxycyline. This is in agreement with the experimental observations; in Fig. 5, the switch off time for the NOPFL network for the different concentration of Doxycycline was estimated from the experimental time-series data (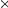 in Fig. 5) (Model simulations and parameter identification).

**Figure 6:**
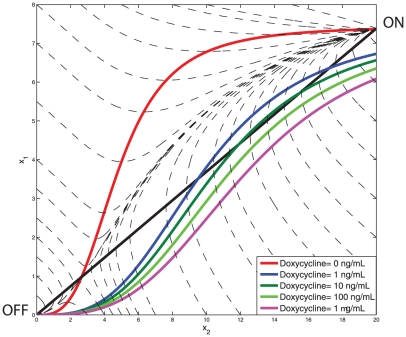
Phase portrait of the PFL model. The tTA-d2EYFP mRNA concentration (y axis) has been plotted against tTA protein concentration (x axis). Varying Doxycycline concentrations (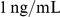 through 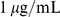) were used to investigate the dependence of the two stable equilibria (“ON” and “OFF” in the graph) on the amplitude of the input. The shape and dimensions of the two basins of attraction (the set of initial conditions ending up in one of the two stable steady states) can be studied with the same technique: in this figure the grey shaded area represents the basin of attraction of the “OFF” equilibrium for Doxycycline = 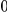 nM.

On the other hand, the PFL network has a very different behaviour, as can be seen in Fig. 5. Specifically, for a range of Doxycycline concentrations, the PFL 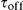 is considerably longer (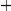 in Fig. 5) that the NOPFL counterpart, which again is in agreement with the experimentally observed behaviour (Material and Methods). In order to investigate the origin of the observed dynamical behavior of the PFL circuit, we analysed the phase portrait associated to the *d2EYFP* 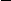 *tTA mRNA* and the tTA protein, which allows to directly observe trajectories of two state variables at once. Moreover, by imposing the steady-state conditions (i.e. 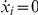), we can derive nullclines, as well as, the their intersection points, which correspond to the equilibrium points of the network. In Fig. 6 the nullclines for different Doxycycline concentrations are shown. When no Doxycycline is present, two stable points (OFF and ON) and one unstable equilibrium points coexist in the same phase portrait, thus providing evidence for the bistability of the PFL network, a shared property among positive feedback loops [21]. However, as Doxycycline concentration increases, the bistability is lost (Fig. 6), and the only possible equilibrium point is the “OFF” state.

## Discussion

We have demonstrated, in a mammalian experimental system, that a transcriptional positive feedback loop can slow down the “switch off” times, as compared to an equivalent network without auto
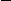
regulation.

The reason for a cell to “choose” a PFL control strategy for transcriptional regulation, rather than the NOPFL strategy, could be due to the intrinsic robustness of this approach to transient activation of the network. For example, in a signalling pathway, a ligand (equivalent to Doxycycline in our PFL) could cause a transcription factor to stop transcribing itself, as well as, a set of target genes, to initiate a specific response. However, in order for the pathway not to respond to a transient concentration of the ligand, the PFL strategy has to be chosen, otherwise the response would start immediately (NOPFL case). Moreover, the response time of the PFL network can be modulated by the ligand concentration, if this is really high, the system will switch off as quickly as possible (Fig. 5), alternatively the ligand can be present at low, or medium, concentration, but it should persist for a long time, in order for the pathway to respond. This kind of behaviour has been recently described as “persistence detection” in cellular signal processing to indicate the ability of the genetic circuit to distinguish between transient and persistent signals [22].

Interestingly, it has been shown in E. coli [23] that the transcriptional negative feedback loop (NFL) has an opposite effect, that is, it can significantly speed up the rise-times of transcription, but has very little effect on the switch-off times. From numerical simulations, we verified that for the PFL the slow down effect is only in the switch off times, whereas rise-times are barely affected (data not shown).

The duality between positive and negative feedback has been predicted in a biological setting [5], and it is a well established concept in “control engineering”, a branch of engineering which deals with the design of automated mechanisms to control a variable of interest (the altitude of an airplane, or more simply, the temperature of a room via thermostat) [24]. Specifically, the negative feedback loop is a classic control engineering approach to speed up the response times of a sytem, thus quickly achieving a desired value of a variable of interest. Positive feedback loops, instead, can slow down the response of the system to external input, and are used by control engineers to build “memory” elements, also known as switches, which are able to be in one of two steady-states (OFF or ON), and which are robust against unwanted transient perturbations that may inadvertently switch off (or on) the system.

We indeed verified that the PFL can exhibit bistability for zero or low concentrations of Doxycyline (Fig. 6). A bistable genetic network will cause a population of cells to divide in two sub-populations, each in one of the two possible states (OFF or ON). In yeast, this has been experimentally verified using a simple PFL based on the rtTA system [21]. In our mammalian PFL, this behaviour was not detected experimentally (FACS analysis data now shown). This can be easily explained by observing that the PFL model is bistable but the basin of attraction of the OFF equilibrium point (Fig. 6) is much smaller as compared to that of the ON state, when no Doxycycline is present. Therefore, just few cells will be in the OFF state and these will not be enough to be significantly detected experimentally. We predict however that for intermediate concentration of Doxycycline (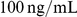 in Fig. 6) the basin of attraction will be comparable and bistability should be detected experimentally. We believe our work can be instrumental to characterise the behaviour of naturally occurring regulatory pathways in mammalian cells and to prove that, despite the complexity of these pathways, they may be understood relying on the knowledge of the behaviour of simplified regulatory network as the one here reported.

## Materials and Methods

### Experimental procedure: Construction of the circuit

To implement the gene circuit in a lentiviral vector, we used the ViraPower Promoterless Lentiviral Gateway Expression System (Invitrogen) which takes advantage of the site-specific recombination properties of bacteriophage lambda, making the transfer of single DNA sequences faster than the usual cloning strategies.

The pMAtTA-IRES-EGFP vector containing the transactivator tTA, the IRES element and the enhanced green fluorescent protein (EGFP) was synthesised by GENEART together with the recombination sites. The d2EYFP was amplified from pd2EYFP-1 (Clontech) by PCR with a forward primer containing a NheI recognition sequence (5′-CATGGCTAGCATGGTGAGCAAGGGCGAGGAG-3′) and a reverse primer containing an EcoRV recognition sequence (5′- ATTCGATATCAGTCGCGGCCGCATCTACA-3′). The PCR product and pMAtTA-IRES-EGFP were then digested with NheI-EcoRV restriction enzymes and the d2EYFP ligated in place of EGFP, generating a new vector termed pMAtTA-IRES-d2EYFP. The pMAtTA-IRES-d2EYFP was then linearised with the AseI restriction enzyme and recombined with the pDONR221 (Invitrogen) following the manufacturer instruction. In this way we generated pENTRtTA-IRES-d2EYFP vector with specific recombination sites.

The *CMV-TET* promoter was amplified from pTRE2 (Clontech) by PCR. The PCR was performed with the Taq polymerase provided by Invitrogen that adds a single deoxyadenosine (A) to the 3′ ends of PCR products. This allows PCR inserts to ligate efficiently with the pENTR5′-TOPO vector which is supplied linearised with single 3′-deoxythymidine (T) overhangs, obtaining the pENTR5′-TOPO-*CMV-TET* with specific recombination sites.

Finally we performed a recombination reaction between the pENTRtTA-IRES-d2EYFP, pENTR5′-TOPO-*CMV-TET* and the pLenti/R4R2/V5-DEST according to manufacturer instructions.

To generate the lentiviral vector containing the gene expression cassette lacking the positive feedback loop (NOPFL), the d2EYFP was amplified from pd2EYFP-1 with the High-Fidelity Taq Phusion (Fynnzimes) which gives blunted-end PCR product. The forward primer (5′-CACCGCCACCATGGTGAGCAAGGGCGAGGAG-3′) was designed to allow the direct cloning in pENTR directional TOPO vector (Invitrogen), generating the pENTR d2EYFP vector. Then we performed a recombination reaction between the pENTR d2EYFP, pENTR5′-TOPO-*CMV-TET* and the pLenti/R4R2/V5-DEST according to manufacturer instructions.

As suggested by the manufacturer, the lentivirus was then produced in 293FTcells.

### Experimental procedure: Cell culture, lentiviral transduction, switch-off experiment

293FT cells were maintained at 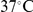 in a 5% CO2-humidified incubator, and cultured in DMEM (GIBCO BRL) supplemented with 10% heat-inactivated fetal bovine serum (FBS) (Invitrogen), 1% L-glutamine, 1% MEM Non-Essential Amino Acids, 1% MEM Sodium pyruvate and 1% antibiotic/antimycotic solution (GIBCO BRL). CHO cells were maintained at 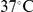 in a 5% CO2-humidified incubator, and cultured in 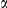-MEM (Sigma) supplemented with 10% heat-inactivated fetal bovine serum (FBS) (Invitrogen), 1% L-glutammine and 1% antibiotic/antimycotic solution (GIBCO BRL). CHO AA8 TET-OFF cell line (Clontech) was maintained 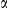-MEM (Sigma)supplemented with 10% TET system approved FBS (Invitrogen), 4 mM L-glutamine, 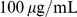 G418 (Sigma), and 1% antibiotic/antimycotic solution (GIBCO BRL).

To transduce cells with the virus produced, 500,000 CHO and CHO AA8 TET-OFF cells were plated and incubated overnight. On the day of transduction the medium was removed and 1 mL of the virus was added to the cells together with polybrene (Invitrogen) to a final concentration of 6 ug/mL. After an overnight incubation the medium containing the virus was removed and replaced with complete culture medium containing Blasticidin (Sigma) to a final concentration of 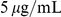 to select for stably transduced cells.

Cells were sorted for fluorescence intensity using a BD FACSAria Cell Sorting System (Becton Dickinson). d2EYFP was excited at 488 nm, and emission was detected using a 525 nm bandpass filter. Serial dilutions of stably transduced cells (up to 0.05 cells/mL) were plated in 96-well microtitre plates, and dilutions containing only one cell per well were selected. Monoclonal colonies were cultured and amplified as described, to obtain monoclonal populations.

For the switch off experiment, 500 stably-integrated-CHO and CHO AA8 TET-OFF cells were plated in chamber slide (lab-Tek) and treated with Doxycycline (Clontech) to a final concentration ranging from 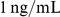 to 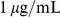). The switch off experiments were repeated twice for the 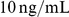 and 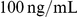 Doxycycline concentrations, while 3 and 5 replicates were obtained for 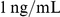 and 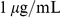. Experiments were performed in parallel for both the PFL and NOPFL cells.

### Experimental procedure: DNA extraction, RealTime PCR

1,000,000 PFL and NOPFL cells were plated in a 6-well multiwell plate to reach a confluence of 80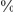 at the moment of the DNA extraction. The day after cells were collected and resuspended in 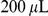 of PBS after centrifugation for five minutes at 300×g. Then the DNA was extracted using the DNeasy Blood and Tissue kit (Qiagen). We compared the DNA levels of *tTA* and *d2EYFP* in NOPFL cells and PFL cells by RealTime PCR following DNA extraction, proving that the both cell populations carry a unique copy of the networks in their genome. Quantitative RealTime PCR reaction were set up in duplicates using the LightCycler 480 SYBR green master mix (Roche) and the amplification was performed using a LightCycler 480 RealTime PCR instrument(Roche). The PCR were carried out using the following primers: d2EYFP forward (5′-acgacggcactcaagacc-3′); d2EYFP reverse (5′-gtcctccttgaagtcgatgc-3′); PFL tTA forward (5′-aaagcagctgaagtgcgagag-3′); PFL tTA reverse (5′-gatggtgctgccgtagttgtt-3′); NO PFL tTA forward (5′-acagcgcattagagctgctt-3′); NO PFL tTA reverse (5′-acctagcttctgggcgagtt-3′). Data analyses were performed using the LightCycler 480 Software(Roche). *GAPDH* DNA levels were used to normalise the amount of DNA and 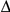
Cts were calculated as the difference between the average *GAPDH* Ct and the average *tTA* and *d2EYFP*.

### Image acquisition and processing

Images were acquired using an inverted epifluorescence microscope (Nikon Eclipse TI-E, Nikon Instruments) equipped with an incubation chamber (H201-OP R2, Okolab), a digital camera (Andor Clara, Andor), a 20× objective (Obj. CFI PF DLL 20× Ph1, Nikon) and a 512-nm/529-nm (B/G/R) d2EYFP-specific excitation/emission filter set. Temperature was maintained at a constant level as the experimental setup required, while CO2 concentration was set to be 5% of the total air volume injected in the incubation chamber. Both phase-contrast images and fluorescent fields were acquired at intervals of 15 minutes. Exposure times for the phase-contrast field was set to 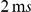 (transmitted light lamp voltage was set to 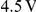) while 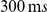 (Intensilight lamp set at 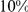 of the maximum power) was chosen as exposure time for the fluorescent images: this choice was meant to prevent photobleaching while optimising the ratio between the quality of the images and reflected-light-induced stress on the cells. Experiments were carried out using NIS-Elements AR v.3.10 644 (Nikon Instruments) software package and the Perfect Focus System (Nikon Instruments) to maintain the same focal plane during the whole duration of the experiment. At the end of the acquisition process, images were extracted as raw data for the fluorescence quantification procedures.

The experiments were set up so that at the beginning of each experiment the first image contained at least 15 cells and no more than 30 cells, to avoid cells exiting the image during the time-lapse experiment due to cell replication and “over-crowding”. Image segmentation was carried out in Mathworks Matlab R2010b (Mathworks Inc.); the algorithm we implemented to quantify fluorescence was meant to distinguish the foreground (living cells) from the background in each image of the bright field. We used morphological operators such as erosion and dilation (*imerode* and *imdilate* functions from the MATLAB image processing toolbox). Thus two binary masks were built in order to compute separately the mean d2EYFP fluorescence of the foreground and the background using an element by element matrix multiplication between the binary images and the fluorescent one. The average fluorescence intensity across the cell population was then computed as the difference between the foreground and the background for each image at each time point (i.e. no single cell fluorescence quantification is performed).

### Experimental procedure: Determination of d2EYFP half-life

To evaluate d2EYFP degradation rate, 500 stably integrated CHO tetOFF cells were plated in chamber slide (lab-Tek) and, after cell adhesion, Cyclohexamide (Sigma, stock dilution 10 mg/ml in sterile water) was added to the medium to a final concentration of 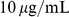, 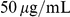, 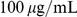 or 500 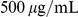. Temperature was maintained at 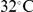. Image acquisition and analysis was performed as described above. The experimental data were fitted into an exponential curve using the curve fitting tool (cftool) from Matlab 2010b, and the degradation coefficient 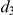 was used to obtain the half-life (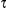) of the d2EYFP protein: 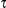 = log(2)/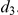


### Model simulations and parameter identification

Numerical simulations were run using Matlab 2010b (Mathworks Inc.). We used *ode23s* solver (a detailed discussion of the numerical methods used by *ode23* can be found in [25]). For the parameter identification, we used the PottersWheel toolbox [19] implemented in MATLAB. Two sets of parameters were identified: the dynamical parameters governing the model and a scaling factor meant to approximate the transduction contribution of the microscopy equipment. Since Doxycycline has been only added at time 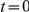 min in our experiment we forced the fitting procedures to start from the model predicted ON steady state.

We defined the following objective function:
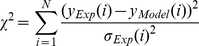
(8)where 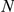 is the number of experimental data points, 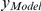 are the predicted values of the mathematical model (using the inferred parameters), 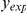 are the experimental data points and 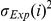 is the sample variance computed over the experimental replicates.

As optimisation algorithm we used Trust Region Method (TRM) in a logarithmic parameter space: at the 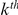 iteration of the optimisation procedure the TRM approximates the shape of the function 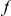 to be minimised with the model 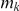 thus trying to solve the following problem:
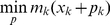
(9)being 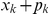 the new parameter vector considered as solution at the 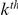 iteration. If the model 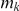 has quadratic form the vector 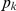 can be obtained by observing that:
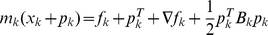
(10)and therefore
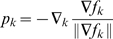
(11)


In order to allow an extensive exploration of the parameters' space, and to avoid local minima, we used a quasi-random number generator routine in PottersWheel [19] to select an initial guess of the parameters' values, and then launched the TRM procedure M times (M = 100 in our settings), requiring the cost function in eq. 8 to be 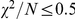 [19].

The values in Table 1 represent parameters for which the cost function (eq. 8) is the smallest across the M runs; whereas the standard devation of each parameter in Table 1 is evaluated by considering all of the M runs.

Moreover, in order to compare switch off times among the different experiments, we computed the 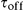 defined as the time the circuit needed to achieve the 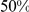 of the mean initial fluorescence 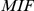 calculated for each experiment as follows:
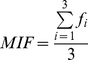
(12)with 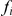 fluorescence of the 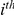 frame in the sequence smoothed by moving average filtering.
